# Microwave-assisted preparation of a silver nanoparticles/N-doped carbon dots nanocomposite and its application for catalytic reduction of rhodamine B, methyl red and 4-nitrophenol dyes

**DOI:** 10.1039/d0ra10679h

**Published:** 2021-01-28

**Authors:** Bhagavanth Reddy G, Ramakrishna Dadigala, Rajkumar Bandi, Kondaiah Seku, Koteswararao D, Girija Mangatayaru K, Ahmed Esmail Shalan

**Affiliations:** Department of Chemistry, Palamuru University Mahabub Nagar Telangana 509001 India bhagavanth.g@gmail.com kgirijamangatayaru@gmail.com; Department of Chemistry, Osmania University Hyderabad Telangana 500007 India; Departement of Engineering (Civil Section), University of Technology and Applied Sciences–Shinas Shinas 324 Sultanate of Oman; Department of Chemistry, Dr. B. R. Ambedkar Open University Hyderabad-500033 India a.shalan133@gmail.com ahmed.shalan@bcmaterials.net; BCMaterials, Basque Center for Materials, Applications and Nanostructures Martina Casiano, UPV/EHU Science Park, Barrio Sarriena s/n Leioa 48940 Spain a.shalan133@gmail.com ahmed.shalan@bcmaterials.net; Central Metallurgical Research and Development Institute (CMRDI) P.O. Box 87 Helwan Cairo 11421 Egypt

## Abstract

In the current work, a silver nanoparticles/nitrogen-doped carbon dots (AgNPs/NCDs) nanocomposite was prepared by a microwave-assisted method that does not require additional reducing or stabilizing agents. Multiple analytical techniques were used to characterize the prepared nanocomposite. The nanocomposite exhibited a surface plasmon resonance (SPR) absorption peak at 420 nm, indicating the development of AgNPs with NCDs. Further, HRTEM results confirmed the formation of the nanocomposite with the appearance of lattice fringes of both materials. Additionally, the nanocomposite did not show any precipitation even after two months of storage. The nanocomposite exhibited high catalytic activity towards the reduction of rhodamine B (RhB, 98.83%), methyl red (MR, 97.14%) and 4-nitrophenol (4-NP, 99.95%) at ambient temperature. Besides, the kinetic analysis revealed that the reduction reaction followed pseudo-first-order kinetics and the calculated rate constants (*k*) for rhodamine B (RhB), methyl red (MR) and 4-nitrophenol (4-NP) were found to be 0.0296 s^−1^, 0.0233 s^−1^ and 0.029 s^−1^, respectively. Moreover, it is a reusable and stable catalyst for reduction reactions up to five cycles without significant loss in catalytic activity. Finally, a plausible mechanism for the reduction of pollutants is also discussed in detail. As a whole, the prepared nanocomposite might display stunning behaviour for wastewater treatment applications.

## Abstract 2

A silver nanoparticles/nitrogen-doped carbon dots (AgNPs/NCDs) nanocomposite was prepared by a microwave-assisted method, and exhibited high catalytic activity towards the reduction of rhodamine B, methyl red and 4-nitrophenol at ambient temperature.

## Graphical abstract

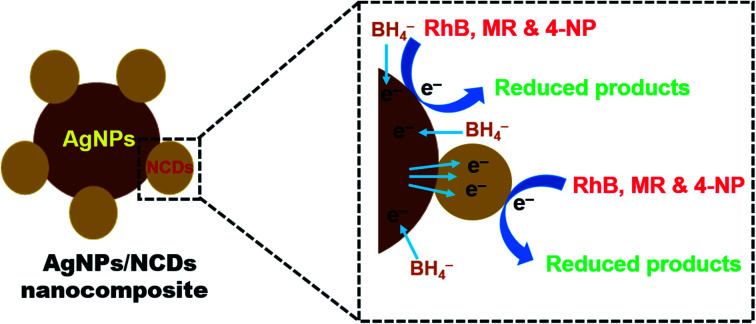

## 1. Introduction

The organic toxins in wastewater from industries such as the pharmaceutical, paper, leather, textile, and plastics industry are of serious concern because of their adverse effects on the ecological system. These organic pollutants are highly carcinogenic and toxic and can be harmful to human health and the environment.^1–7^ Thus, to get rid of this hidden risk, the development of efficient treatment technology is necessary to fulfill the ever-increasing demand for sustainable water. Several conventional approaches such as chemical, physical and biological processes have been tested to eliminate these pollutants.^8–12^ The chemical reduction of these pollutants in the existence of NaBH_4_ (as reducing agent) and metal nanoparticles (as a catalyst) is one of the most commonly used techniques.^8,13,14^ Noble metal nanoparticles (silver, gold, palladium, and platinum) have attracted significant attention as a result of their unique optical and physical features as well as their large surface to volume ratio, which makes them applicable in different fields such as biomedical, electronics, photonics, sensing and catalysis.^15–20^ Among them, mainly silver nanoparticles (AgNPs) have attracted considerable attention because of their high electrical conductivity, cost-effectiveness and antimicrobial activity as well as showing high catalytic efficacy in various chemical reactions such as alkane and alkene oxidation, reduction of dyes, hydrogenation of 4-NP and as intermediates in pharmaceutical reactions.^21–25^

A broad series of methods like chemical, physical, laser ablation, hydrothermal, sol–gel, ultrasonication, microwave irradiation and electrochemical have been developed to obtain AgNPs, and additional significant efforts have been dedicated to controlling their shape as well as size that can significantly influence the features of AgNPs.^23,26–28^ Among all the synthetic strategies, chemical pathways can govern both the shape and size of the AgNPs, but most of the chemical methods are depending on the reduction of AgNO_3_ with a strong reducing agent like hydrazine hydrate, sodium citrate and sodium borohydride.^29,30^ These reagents are exceedingly toxic, sensitive and have biological risks. To control their shape and size in nanoparticle synthesis, polymers, DNA and other efficient molecules are used as stabilizers.^31–34^ But, these molecules on the surface of nanoparticles always hinder the reactants from approaching the active sites of nanoparticles and restricting their properties and lowering the activities. Hence, some procedures such as thermal treatment and washing have been established to eradicate molecules on nanoparticles' surface. Though, removing the molecules on nanoparticles leads to the agglomeration of nanoparticles, changing their properties.^35,36^ To overcome these problems, it is necessary to prepare surfactant-free nanoparticles with controlled size and shape. In recent times, carbon nanoparticles have been proved to retain better advantages compared to the traditional organic surfactants.^37,38^ Thus, we have confidence in the phenomenon intended that the nanoparticles fabricated with carbon nanoparticles may have superior features. N-doped carbon dots (NCDs), a new class of carbon nanoparticles have recently been used to synthesize AgNPs and AuNPs. Li-Ming Shen and co-workers reported growth of the AgNPs with NCDs, Peihui Luo *et al.*, described the preparation of gold@carbon dots nanocomposite, B Sinduja and Abraham had prepared silver nanoparticles capped with NCDs.^39–41^ From this literature, we understood that the CDs are efficient for preparation of metal nanoparticles with controlled size. Moreover, presence CDs can enhance the adsorption of organic pollutants on the catalyst surface due to their conjugative structure, which leads to the fast reduction of pollutants. From these advantages we have prepared AgNPs/NCDs nanocomposite by using NCDs as reducing and stabilizing agents.

Herein, we report a microwave-assisted synthesis of silver nanoparticles/nitrogen-doped carbon dots (AgNPs/NCDs) nanocomposite. Here, NCDs can directly reduce AgNO_3_ to AgNPs in the absence of any harmful reducing and stabilizing agent. These NCDs were prepared from *Lantana camara* fruit extract and ethylenediamine in our previous work. Further, the effect of varying concentrations of AgNO_3_ and microwave reaction time on the synthesis was also studied. Various analytical techniques were applied to investigate the optical, structural as well as morphological features of the as-prepared nanocomposite. The catalytic activity of AgNPs/NCDs nanocomposite in the reduction of rhodamine B (RhB), methyl red (MR) and 4-nitrophenol (4-NP) in the existence of NaBH_4_ in aqueous media has also been reported. Most importantly, only a slight loss is observed in AgNPs/NCDs nanocomposite catalytic activity even after several recycles. To the best of our knowledge, this is the first report on the fabrication of AgNPs/NCDs nanocomposite by microwave irradiation method and as a catalyst for the reduction of RhB, MR and 4-NP dyes in wastewater.

## 2. Experimental

### 2.1. Materials

AgNO_3_, NaBH_4_ purchased from Sigma-Aldrich; RhB, MR and 4-NP from S. D. Fine Chemicals, India. All chemicals were applied as received and double-distilled (DD) water applied for all reactions.

### 2.2. Synthesis of AgNPs/NCDs nanocomposite

NCDs were prepared as per our previously reported method.^42^ Then AgNPs/NCDs nanocomposite was prepared by microwave method. In brief, 500 μL of AgNO_3_ solution was added to the NCDs (1 mL, 1 mg mL^−1^) solution, then the volume was prepared to 4 mL using DD water. After that, the solution was exposed to microwave irradiation for 2 min at 450 W power, and the formed light brown colour solution indicated the successful synthesis of AgNPs/NCDs nanocomposite. Finally, the nanocomposite solution was centrifuged at 8000 rpm for 10 min, and then the collected pellet was dried at 80 °C. Meanwhile, AgNPs/NCDs nanocomposite synthesis was also optimized at diverse concentrations of AgNO_3_ (0.1 to 2 mM) with different microwave irradiation times (30 s to 120 s).

### 2.3. Characterization

Several analytical techniques confirm the prepared nanocomposite. Shimadzu, UV-2600 spectrophotometer was used to measure the optical properties. XRD (Rigaku Miniflex 600) was used to find the crystal structure of the nanocomposite. Shimadzu IR Prestige-21 spectrophotometer was employed to detect the functional groups that participated in metal reduction and stabilization. TEM (JEOL JEM 2100) was applied to measure the size in addition to the morphology of the prepared nanocomposite. Besides, XPS analysis was achieved using a Kratos AXIS Ultra spectrometer *via* Al Kα (1486.71 eV). DLS (Malvern instrument Ltd, Malvern, UK) was applied to define the zeta potential of the synthesized nanocomposite.

### 2.4. Catalytic activity of the AgNPs/NCDs

Catalytic capability of synthesized nanocomposite was considered to reduce RhB, MR and 4-NP using the NaBH_4_ as a hydrogen generator. RhB or MR solution (2 mL of 1 mM) and NaBH_4_ solution (1 mL of 10 mM) were taken in a test tube, and then DD water was added to it to make the total reaction mixture 10 mL. After that, 3 mL reaction mixture was transferred into a cuvette, followed by 8 mg of as-prepared AgNPs/NCDs nanocomposite was added to it. Then the UV-visible spectra were recorded at every 1 minute to monitor the reduction reaction. Further, the 4-NP reduction was also accomplished. In a typical procedure, 1.5 mL of 0.2 mM 4-NP solution and 1 mL of 2 mM NaBH_4_ was taken in a cuvette, then 8 mg of AgNPs/NCDs nanocomposite was added to it. The absorption spectra were recorded at every 1 minute to monitor the reduction reaction. Without the incorporation of catalyst control experiments were also carried out. However, the reduction reaction kinetics was also studied through observing the depletion in the absorbance.

## 3. Results and discussion

**Scheme 1:**
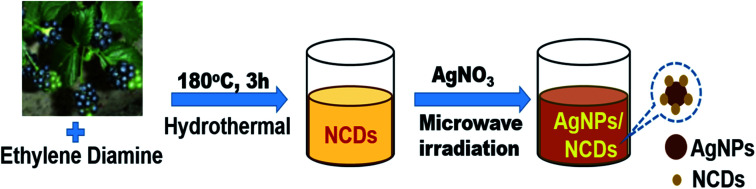
Schematic illustration for the synthesis of AgNPs/NCDs nanocomposite.

Initially, highly fluorescent NCDs were synthesized by one-step hydrothermal carbonization of *Lantana camara* berries and ethylene diamine; the synthesis and characterization were reported in our previous publication.^42^ In this work, the prepared NCDs were mixed with AgNO_3_ solution, and the mixture was exposed to microwave irradiation for 2 min at 450 W. The resulting reaction mixture colour was altered from light yellow to light brown, representing the creation of AgNPs/NCDs nanocomposite (Scheme 1), which is further confirmed by various analytical techniques.

**Fig. 1:**
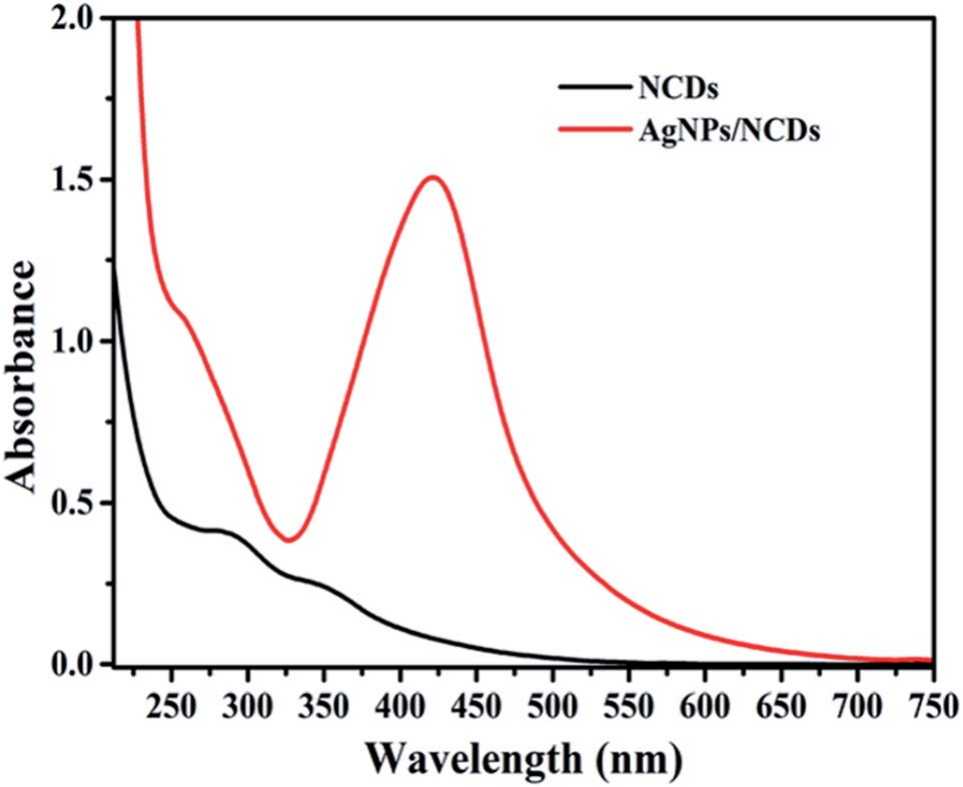
UV-vis absorption spectra of NCDs and AgNPs/NCDs nanocomposite.

**Fig. 2:**
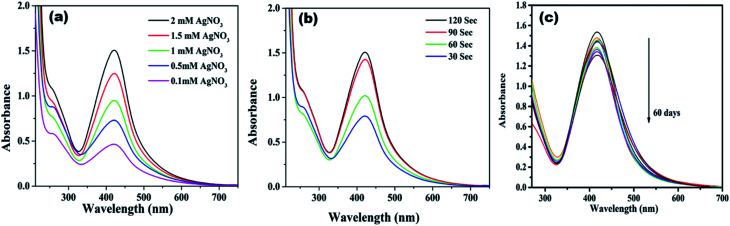
UV-vis absorption spectra of AgNPs/NCDs nanocomposite synthesized at (a) different concentration of AgNO_3_, and (b) different microwave irradiation time; (c) UV-vis spectra of AgNPs/NCDs depicting their stability for 2 months.

Fig. 1 displays the UV-visible spectra of NCDs and AgNPs/NCDs nanocomposite. The pure NCDs show absorption peaks at 285 nm and 356 nm corresponding to the π–π* and n–π* transitions.^42^ After addition of AgNO_3_, a new peak appeared at 420 nm consistent with the surface plasmon resonance of AgNPs.^32^ However, the absorption peaks of NCDs disappeared. The effects of different concentrations of AgNO_3_ and microwave irradiation time were also optimized on nanocomposite formation (Fig. 2). The concentration range of AgNO_3_ used was 0.1–2 mM possessed the other parameters constant, and the reaction was examined *via* evaluating the absorbance of a reaction mixture. The corresponding absorbance changes were shown in Fig. 2a; it is seen that increasing the concentration of AgNO_3_ resulted in the formation of more number of AgNPs/NCDs nanocomposite. Further, we also examined the effect of microwave irradiation time by keeping other parameters constant. The reaction was monitored between 30 to 120 s at 450 W and the resulted absorption spectra depicted in Fig. 2b; shows that gradual increase in the microwave irradiation time up to 120 s, increased the amount of AgNPs/NCDs nanocomposite formation.^13,23,43^ Under these conditions, the yield of the nanocomposite was found to be 5.8%. Besides, Fig. 2c shows AgNPs/NCDs nanocomposite stability, wherein no significant change was observed in peak intensity even after storage for 60 days. This indicates that the NCDs shield and make the AgNPs become stable from accumulation in the aqueous medium.

**Fig. 3:**
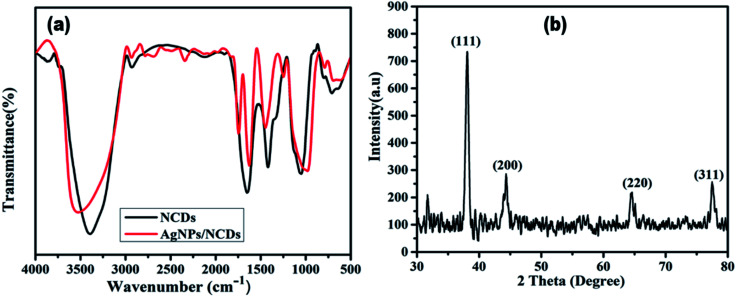
(a) FTIR spectra of NCDs and AgNPs/NCDs nanocomposite. (b) XRD pattern of AgNPs/NCDs nanocomposite.

To identify the surface functional groups of NCDs that are responsible for the reduction and stabilization of the AgNPs, FTIR analysis was accomplished. Fig. 3a demonstrates the FTIR spectrum of the NCDs and AgNPs/NCDs nanocomposite. In NCDs, the broad peak centred at 3395 cm^−1^ is accredited to the stretching vibrations of O–H/N–H groups. However, the absorption peaks at 2935, 1659, 1411 and 1053 cm^−1^ suggest the existence of the C–H, C=O, N–H and C–O groups,^42^ respectively. In the FTIR spectra of AgNPs/NCDs nanocomposite, absorption bands are observed at 3547, 2930, 1756, 1604, 1438 and 963 cm^−1^. Comparison of NCDs and AgNPs/NCDs FTIR spectra, reveals that the peak positions of the nanocomposite were shifted from 3395 to 3547 cm^−1^, 1659 to 1604 cm^−1^ and 1411 to 1438 cm^−1^, and the intensities of peaks were decreased, due to the interaction between the NCDs and AgNPs, confirming the formation of AgNPs/NCDs nanocomposite. Additionally, a new peak at 1745 cm^−1^ appeared in AgNPs/NCDs spectrum, which corresponds to symmetrical stretching of carboxylate groups.^39,41,44,45^ From the FTIR spectrum, we can conclude that NCDs have abundant –NH_2_, carbonyl and –OH functional groups on the surface that acted as electron donors for the reduction of Ag^+^ to elemental Ag^0^.^46,47^

The crystalline structure of the synthesized AgNPs/NCDs nanocomposite was identified through XRD analysis. As exposed from Fig. 3b, the diffraction peaks located at 38.14°, 44.54°, 64.61° and 77.51° can be indexed to the (111), (200), (220) and (311) Braggs reflection of face centred cubic structure of metallic silver respectively (JCPDS No. 04-0783).^23,28^ XRD investigation suggested that the synthesized AgNPs/NCDs nanocomposite was composed of crystalline silver.

**Fig. 4:**
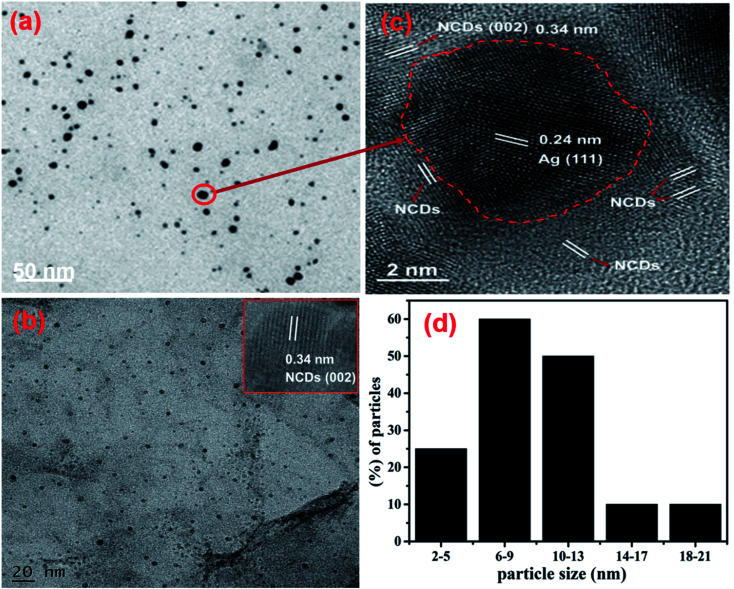
(a) TEM image and (b) HRTEM image of the AgNPs/NCDs nanocomposite. (c) TEM image of NCDs and its HRTEM image in inset; (d) size distribution histogram of the AgNPs/NCDs nanocomposite.

TEM analysis was supported to identify the shape in addition to the size of the fabricated AgNPs/NCDs nanocomposite (Fig. 4). As shown in Fig. 4a, the AgNPs/NCDs nanocomposite has a spherical shape and is well dispersed. The dark spots appeared in the TEM image mainly due to high crystallinity of AgNPs, but NCDs combined with AgNPs did not appear clearly in TEM image, because of the amorphous nature and low contrast of NCDs. Therefore, further we provided the HRTEM image for confirming the formation of composite between NCDs and AgNPs. In HRTEM image (Fig. 4b) the characteristic lattice fringe spacing of 0.24 nm and 0.34 nm was observed, which is ascribed to the (111) plane of AgNPs and (002) plane of NCDs, respectively.^48,49^ In addition, NCDs are clearly found on the surface of AgNPs that confirmed the formation of AgNPs/NCDs nanocomposite. Moreover, for comparison we provided TEM image of NCDs (Fig. 4c) and its HRTEM image (shown in inset). From Fig. 4c it was observed that the NCDs appear as low contrast spots and from the inset NCDs have lattice fringe spacing of 0.34 nm (002). The size distribution histogram (Fig. 4d) demonstrates that the nanocomposite's average diameter is 9 ± 3 nm.

**Fig. 5:**
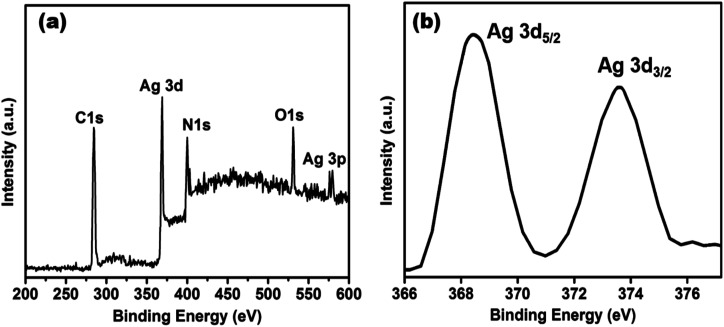
(a) XPS survey spectrum of AgNPs/NCDs nanocomposite, (b) high-resolution spectrum of Ag 3d of AgNPs/NCDs nanocomposite.

XPS measurement was checked to ascertain further the surface composition of the as-prepared AgNPs/NCDs nanocomposite (Fig. 5). As found in Fig. 5a, the XPS survey spectrum of the nanocomposite; it clearly displays the peaks at around 285, 370, 400, 531 and 580 eV, which are corresponding to C 1s, Ag 3d, N 1s, O 1s and Ag 3p state, respectively.^48^ The strong C 1s, N 1s and O 1s peaks were corresponding to the NCDs prepared from the *Lantana camara* and ethylenediamine as reported in our previous article.^42^ Furthermore, the high-resolution spectrum of Ag 3d (Fig. 5b) displayed two different peaks at 368.2 and 374.2 eV, which were accredited to the binding energies of Ag 3d_5/2_ and Ag 3d_3/2_, respectively. It is indicating the metallic state of formed silver nanoparticles.^48^

**Fig. 6:**
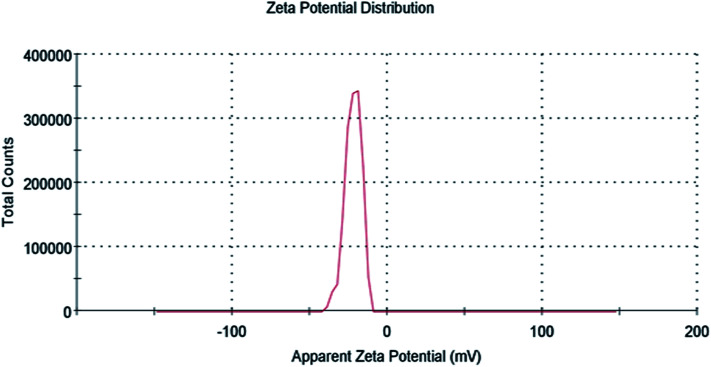
Zeta potential distribution of AgNPs/NCDs nanocomposite.

Zeta potential gives information about the state of the surface charge and predicts the colloidal system's long-term stability.^44^ To further confirm AgNPs/NCDs nanocomposite formation, the zeta potential values of NCDs and AgNPs/NCDs were measured (Fig. 6). NCDs zeta potential was −11 mV, and AgNPs/NCDs composite was −22.1 mV, respectively. The negative charge on NCDs surface was due to nitrogen and oxygen functional groups.^42^ In the case of AgNPs/NCDs nanocomposite, the increased zeta potential was due to the attachment or binding of more than one NCD on AgNPs surface. Thus, the increase in the zeta potentials indicates that the AgNPs/NCDs nanocomposite was relatively stable and NCDs assist as reducing and capping agents for AgNPs/NCDs.^41^

### 3.1. Mechanism of AgNPs/NCDs nanocomposite formation

Several publications concluded that the plant-based materials are accountable for reducing metal ions into metallic nanoparticles accompanied by capping to inhibit agglomeration.^32^ In this work, we used a plant material derived NCDs to reduce and stabilise AgNPs. In our previous publication, we have prepared NCDs from *Lantana camara* berries and ethylenediamine as carbon and nitrogen sources, respectively. Several analytical techniques characterized the prepared NCDs. According to FTIR and XPS studies, NCDs have numerous functional groups, for instance, hydroxyl, amino, carbonyl and carboxylate groups.^42^ Under microwave irradiation, these functional groups combine with Ag^+^ ions and reduce into AgNPs, which further prevent agglomeration of nanoparticles. MWI generates localized superheating which results in the rapid reaction. In the FTIR analysis, when the AgNPs/NCDs nanocomposite was formed by using NCDs functioning as the reducing and capping agent, it is depicted that the peak intensities of N–H/O–H and C=O are found to be decreased in comparison with the NCDs and appearance of a new band for –COO^−^ group. The increased zeta potential also further confirmed the formation of the nanocomposite. These results demonstrate that the N–H/O–H and C=O groups of NCDs were broken to carboxyl groups after forming AgNPs/NCDs nanocomposite.^50,51^

### 3.2. Catalytic activity of AgNPs/NCDs nanocomposite

The catalytic efficacy of AgNPs/NCDs nanocomposite was evaluated towards the reduction of model contaminants such as rhodamine B (RhB), methyl red (MR) and 4-nitrophenol (4-NP). The reaction was carried out at an ambient temperature and was monitored by UV-visible spectrophotometer.

**Fig. 7:**
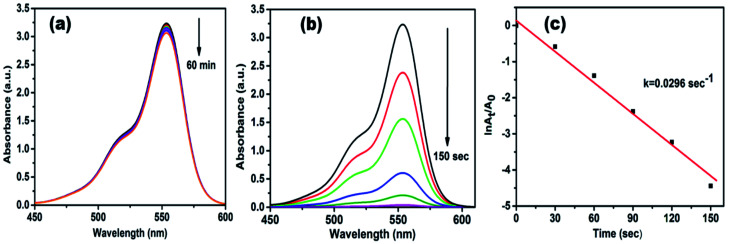
UV-vis absorption spectra of (a) catalytic reduction of RhB in the presence of NaBH_4_ and in the absence of catalyst; (b) time-dependent absorption spectra of RhB in the existence of both NaBH_4_ and AgNPs/NCDs; (c) plot of ln(*A*_*t*_/*A*_0_) against time for reduction of RhB dye.

Rhodamine B (RhB) is a cationic dye, which is widely used in many industries, and it is hazardous as well as mutagenic in nature. The aqueous solution of RhB shows an absorption peak at 554 nm. The variation in the intensity of peak at 554 nm was used to observe the whole reduction process. As shown in Fig. 7a, up to 60 min, a small decrease was observed in peak intensity only in the presence of NaBH_4_, while in the attendance of both AgNPs/NCDs nanocomposite and NaBH_4_, the absorption peak intensity was gradually decreased and finally 98.83% of RhB was reduced in 150 s (Fig. 7b). Here, the taken NaBH_4_ concentration was greater than the RhB concentration. Hence, the rate of the reaction depended on RhB concentration only.^10,13^ The linear plot of ln(*A*_*t*_/*A*_0_) *versus* time indicates that the reduction reaction followed the pseudo-first-order kinetics, and the calculated rate constant (*k*) was 0.0296 s^−1^ (Fig. 7c).

**Fig. 8:**
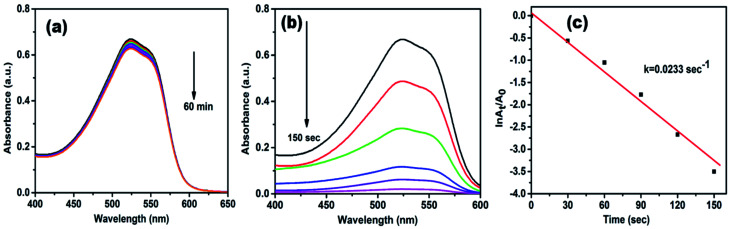
UV-vis absorption spectra of (a) control experiment of MR (b) time-dependent absorption spectra of MR over AgNPs/NCDs nanocomposite and NaBH_4_; (c) plot of ln(*A*_*t*_/*A*_0_) against time for reduction of MR dye.

Methyl red (MR) is another common water pollutant and widely found in many industrial wastewaters. Hence, removal of it from industrial effluents is a primary task nowadays. MR showed absorption peaks at 524 nm in the aqueous medium, and alteration in absorption intensity was employed to study the whole reduction process. Fig. 8a displays UV-vis absorption spectra of MR with NaBH_4_ only, but even after 60 min, there is no noteworthy change in peak intensity, it indicates the reduction process is prolonged. However, in the presence of AgNPs/NCDs nanocomposite in MR and NaBH_4_ solution, a gradual decrease in the intensity was detected, and the reduction reaction was accomplished (97.14%) within 150 s (Fig. 8b). Additionally, Fig. 8c shows a linear correlation between ln(*A*_*t*_/*A*_0_) and time and the calculated rate constant (*k*) from the slope was 0.0233 s^−1^.

**Fig. 9:**
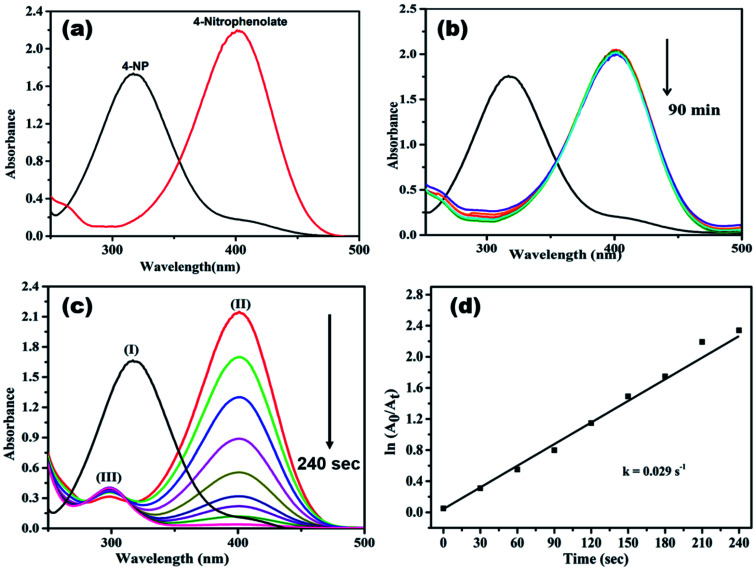
UV-visible absorption spectra of (a) 4-nitrophenol and 4-nitrophenolate; (b) control experiment of 4-nitrophenol; (c) reduction process of 4-NP in the presence of AgNPs/NCDs nanocomposite and NaBH_4_ [(I) 4-NP, (II) 4-nitrophenolate and (III) 4-aminophenol]; (d) plot of ln(*A*_*t*_/*A*_0_) *vs.* time for the reduction of 4-NP dye.

Generally, 4-NP (light yellow colour) shows absorption peak at 318 nm, when NaBH_4_ is added to it, the absorption peak is shifted to 400 nm (Fig. 9a), due to the formation of 4-nitrophenolate ion (deep yellow).^13,15,52^ In a control experiment it was noticed that the absorbance peak at 400 nm did not change even after 90 min (Fig. 9b), it indicates the reduction of 4-NP was not proceeded without adding catalyst.^16,18,37^ When AgNPs/NCDs nanocomposite was added to 4-NP + NaBH_4_ solution the absorption peak considerably decreased within 240 s, meanwhile a new peak found around 297 nm, and the peak intensity gradually increased with time, it corresponds to the 4-aminophenol (4-AP) product (Fig. 9c)^13,15,22^ and about 99.95% of 4-NP was reduced to 4-AP. As shown in Fig. 9d, a linear correlation was found between ln(*A*_*t*_/*A*_0_) and time and the calculated rate constant (*k*) was about 0.029 s^−1^.

### 3.3. Catalytic mechanism of AgNPs/NCDs nanocomposite

**Fig. 10:**
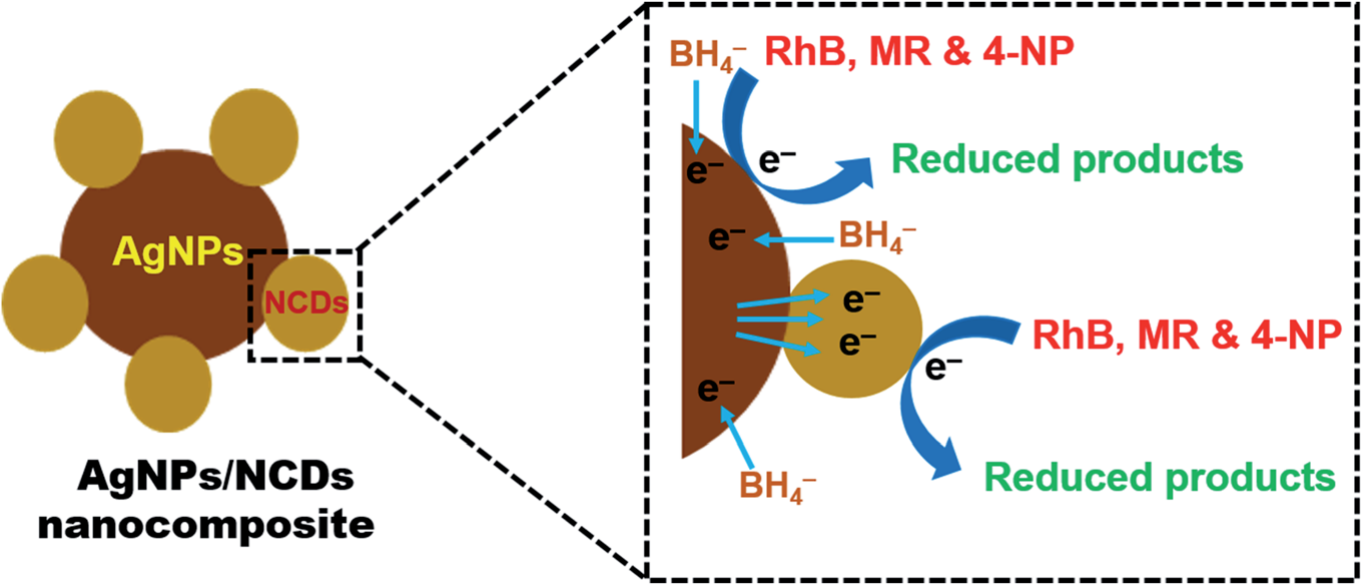
Schematic representation of a plausible mechanism for the reduction of RhB, MR, and 4-NP dyes in the presence of AgNPs/NCDs nanocomposite and NaBH_4_.

From the above results, it was observed that the reduction reaction was completely negligible in the presence of NaBH_4_ only. But in the existence of both nanocomposite and NaBH_4_, the reduction reaction was taking place and completed within a few seconds. From these observations, it was concluded that the AgNPs/NCDs nanocomposite played a role of catalyst for RhB, MR and 4-NP reduction. Based on this, a reasonable mechanism was proposed for the reduction of these pollutants (Fig. 10). While in the presence of nanocomposite, both BH_4_^−^ ions and pollutant molecules co-adsorbed on the surface of nanocomposite. As the pollutants used in the present study have aromatic rings in their structures they get readily adsorbed on the catalyst surface due to the π–π interaction between NCDs conjugated structure and aromatic rings of pollutants.^48^ During the reduction reaction, the electrons transfer from the BH_4_^−^ ion to direct AgNPs and through NCDs to AgNPs, at the same time the formed electrons in AgNPs transfer to surface functional groups of NCDs. Then these electrons transferred to the adsorbed pollutants; further, these pollutants were successively reduced.^13,15,35^

### 3.4. Recyclability of catalyst

**Fig. 11:**
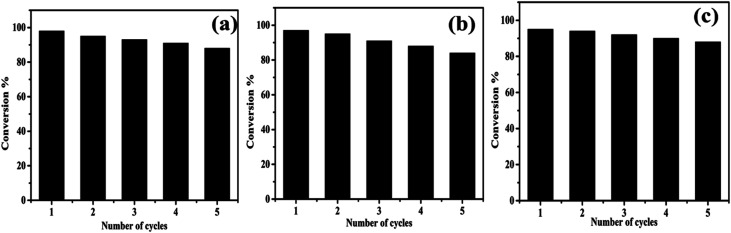
Reusability of formed AgNPs/NCDs nanocomposite towards the reduction of (a) RhB, (b) MR and (c) 4-NP dyes.

Further, we studied the reusability of the catalyst because it is a vital parameter in industries. To explore the stability of formed nanocomposite, recycling experiments were executed by reducing RhB, MR, and 4-NP under NaBH_4_. After each cycle, the catalyst was recovered through centrifugation and washed and oven-dried for the next cycle. Thus, the recovery and recycling experiments were implemented for 5 cycles. As shown in Fig. 11, little loss in the catalytic activity was detected up to five cycles, which implies the high stability of formed nanocomposite during the reduction process.

## 4. Conclusion

In summary, we have prepared AgNPs/NCDs nanocomposite by a microwave irradiation method. Characterization results revealed that the formed nanocomposite showed SPR absorption band at 420 nm, it corresponds to the AgNPs, and further HRTEM results confirmed the nanocomposite formation, due to the existence of lattice fringes of both materials. After storage for two months, the formed nanocomposite was not shown in any deposition in the bottom of the test-tube. The catalytic activity of formed nanocomposite was studied by a reduction reaction of various pollutants like RhB, MR and 4-NP under NaBH_4_. These pollutants' reduction reactions followed the pseudo-first-order kinetics, with rate constants 0.0296 s^−1^, 0.0233 s^−1^ and 0.029 s^−1^, respectively. Further, the nanocomposite showed good recycling ability for five cycles with only little loss in catalytic activity. Therefore, these AgNPs/NCDs nanocomposites will show promising environmental safety and the treatment of several industrial effluents.

## Author contributions

B. R. G. helps in preparing material, characterized them by different characterization techniques and writing the manuscript. R. D., R. B. and K. S. helps in investigation, methodology, data curation and analytic characterization. K. D. and G. M. K. contributed in the characterization of the obtained materials and discussed the results. Furthermore, B. R. G. and A. E. S. designed the research, contributed to supervising the work, discussed the results and wrote the manuscript. All the authors participated in writing, editing and revising the manuscript.

## Conflicts of interest

The authors declare that they have no known competing financial interests or personal relationships that could appear to influence the work reported in this paper.

## Supplementary Material
